# Exoscopic Microsurgery: A Change of Paradigm in Brain Tumor Surgery? Comparison with Standard Operative Microscope

**DOI:** 10.3390/brainsci13071035

**Published:** 2023-07-06

**Authors:** Andrea Di Cristofori, Francesca Graziano, Chiara Benedetta Rui, Paola Rebora, Diego Di Caro, Gaia Chiarello, Giovanni Stefanoni, Chiara Julita, Santa Florio, Davide Ferlito, Gianpaolo Basso, Giuseppe Citerio, Paolo Remida, Giorgio Carrabba, Carlo Giussani

**Affiliations:** 1 Department of Medicine and Surgery, University of Milano-Bicocca, Ospedale San Gerardo, Piazza Ateneo Nuovo, 120126 Milan, Italydiego.dicaro@irccs-sangerardo.it (D.D.C.); gianpaolo.basso@unimib.it (G.B.); giuseppe.citerio@unimib.it (G.C.);; 2 Neurosurgery, Fondazione IRCCS San Gerardo dei Tintori Via G.B. Pergolesi 33, 20900 Monza, Italy; 3 Bicocca Bioinformatics, Biostatistics and Bioimaging Centre—B4, School of Medicine and Surgery, University of Milano-Bicocca, Piazza Ateneo Nuovo, 120126 Milan, Italy; 4 Pathology, Fondazione IRCCS San Gerardo dei Tintori, Via G.B. Pergolesi 33, 20900 Monza, Italy; 5 Neurology, Fondazione IRCCS San Gerardo dei Tintori, Via G.B. Pergolesi 33, 20900 Monza, Italy; 6 Radiotherapy, Fondazione IRCCS San Gerardo dei Tintori, Via G.B. Pergolesi 33, 20900 Monza, Italy; 7 Neuroradiology, Fondazione IRCCS San Gerardo dei Tintori, Via G.B. Pergolesi 33, 20900 Monza, Italy; 8 Neurointensive Care Unit, Department of Neuroscience, Fondazione IRCCS San Gerardo deiTintori, Via G.B. Pergolesi 33, 20900 Monza, Italy

**Keywords:** brain tumors, neurosurgery, glioblastoma, extent of resection, progression-free survival, en bloc resection, perilesional resection, exoscope, operative microscope

## Abstract

Background: The exoscope is a high-definition telescope recently introduced in neurosurgery. In the past few years, several reports have described the advantages and disadvantages of such technology. No studies have compared results of surgery with standard microscope and exoscope in patients with glioblastoma multiforme (GBM). Methods: Our retrospective study encompassed 177 patients operated on for GBM (WHO 2021) between February 2017 and August 2022. A total of 144 patients were operated on with a microscope only and the others with a 3D4K exoscope only. All clinical and radiological data were collected. Progression-free survival (PFS) and overall survival (OS) have been estimated in the two groups and compared by the Cox model adjusting for potential confounders (e.g., sex, age, Karnofsky performance status, gross total resection, MGMT methylated promoter, and operator’s experience). Results: IDH was mutated in 9 (5.2%) patients and MGMT was methylated in 76 (44.4%). Overall, 122 patients received a gross total resection, 14 patients received a subtotal resection, and 41 patients received a partial resection. During follow-up, 139 (73.5%) patients experienced tumor recurrence and 18.7% of them received a second surgery. After truncation to 12 months, the median PFS for patients operated on with the microscope was 8.82 months, while for patients operated on with the exoscope it was >12 months. Instead, the OS was comparable in the two groups. The multivariable Cox model showed that the use of microscope compared to the exoscope was associated with lower progression-free survival (hazard ratio = 3.55, 95%CI = 1.66–7.56, *p* = 0.001). Conclusions: The exoscope has proven efficacy in terms of surgical resection, which was not different to that of the microscope. Furthermore, patients operated on with the exoscope had a longer PFS. A comparable OS was observed between microscope and exoscope, but further prospective studies with longer follow-up are needed.

## 1. Introduction

Neurosurgery is a highly advanced surgery that requires the use of highly technological tools for performing high-precision surgeries [1,2,3]. This has become particularly true after the introduction of the operative microscope in the clinical practice in 1950s [4]. Starting from this period, progress in neurosurgery has been marked by the introduction of technological tools such as neuronavigation on, intraoperative ultrasound, etc.

Glioblastoma multiforme (GBM) is a primary brain tumor of astrocytic origin with highly malignant behavior. It represents the most frequent malignant glioma, accounting for 69% of all primary brain tumors [5,6]. Nowadays, the treatment of GBM requires maximal safe resection followed by adjuvant chemo- and radiation therapy [7,8,9]. In particular, surgical resection still affects prognosis in terms of both progression-free survival (PFS) and overall survival (OS) [10,11,12,13,14,15]. The extent of surgical resection (EOR) depends on several factors: the side and site of the tumor, tumor volume, and the relation of the tumor with eloquent areas and subcortical white matter bundles [10,11,16,17].

In recent years, the introduction of the surgical exoscope is trying to mark a change of surgical paradigm. In fact, the exoscope is an external telescope made of a highly technological camera characterized by the possibility of high magnification and digital elaboration of acquired images [18,19]. The first exoscopes used for surgery were characterized by 2D images that did not allow a stereoscopic perception of the surgical field [20]. In recent years, exoscopes have been equipped with a 3D screen able to deliver a stereoscopic vision of the surgical field [19]. With this technological progress, the use of exoscope presents similar features to the operative microscope [21,22,23]. In this way, it would be useful to better understand advantages and disadvantages of both technologies in order to personalize the surgical setting according not only to individual surgeon preferences but also according to the patients’ needs.

From 2021, in our division, a brand new exoscope (Orbeye™ by Olympus™—Tokyo, Japan) was introduced into clinical practice alongside the standard traditional operative microscope (Leica Microsystems™—Wetzlar, Germany). This event marked a type of revolution in our division that led to a new decision process in the surgical theater when performing neurosurgical procedures.

With our paper, we would like to present the results regarding the comparisons between the use of the operative microscope and the exoscope in a consecutive series of patients operated on for GBM. The terms of comparisons were survival outcome, both as PFS and OS; surgical outcome as percentage of tumor resection; and clinical outcome as Karnofsky performance status and postoperative new deficit. We also would like to share our first impressions during this long period of use of the surgical exoscope. This will help neurosurgeons to better understand the difficulties and advantages that could occur during the use of an exoscope.

## 2. Materials and Methods

### 2.1. Design and Inclusion Criteria

Our retrospective study included all patients operated on for GBM from February 2017 to August 2022 at Fondazione IRCSS San Gerardo deiTintori in Monza, Italy. Follow-up ended 12 May 2023. Each histological diagnosis before 2021 was reviewed and reclassified according the WHO 2021 Classification of Tumors of the Central Nervous System [24]. The series encompassed adult patients (>18 years old), with a minimum of 6-month follow-up at our institution, who underwent a craniotomy for the resection of a primary brain tumor. Only patients with GBM were included in the study.

### 2.2. Exclusion Criteria

Patients who underwent chemo or radiotherapy at other institutions were excluded. We excluded patients operated on for tumors other than GBM. Patients who received a purely bioptic procedure were excluded. All patients without preoperative and postoperative MRI were excluded. All patients not able to sign a consent form were also excluded from this study.

### 2.3. Data Collection

Data collection was performed prospectively and comprised demographic data, including age at diagnosis, sex, side and site of the tumor, pre- and post-operative Karnofsky performance status (KPS), neurological deficits at presentation and/or after surgery, death, and progression status. The cutoff used as comparison for KPS was 70. Use of exoscope or operative microscope and post-operative new deficits were also recorded. Surgeon’s expertise (high and low experience) was defined as more than 15 years of practice on major surgeries (with glioblastoma representing more than 20% of them).

All surgeries were performed with perilesional resection as previously described [16]. Surgery with microscope was performed with the operative microscope Leica M530 OH6 (Leica Mycrosystems^®^, Wetzlar, Germany); surgery with exoscope was performed with Orbeye 3D4K exoscope (Olympus^®^, Tokyo, Japan).

### 2.4. Radiological Data

All patients underwent pre- and post-operative brain MRI in order to assess the extent of surgical resection (EOR). EOR was measured according to Berger et al.(2011) based on pre-operative and post-operative volumetric with T1-weighted gadolinium brain MRIs [13,15].

Preoperative tumor volume, residual tumor volume, and percentage of resection were calculated using BrainLab™ v. 3.0 segmentation software (BrainLab™, Germany). Gross total resection (GTR) was considered when 99% of the tumor volume was removed; subtotal resection (STR) was considered when 98% to 90% of the tumor was removed; and partial resection (PR) when below 90% was removed.

### 2.5. Neuro-Oncological Treatment and Follow-Up

Standard first-line treatment was concomitant chemo-radiation therapy according to the Stupp’s protocol [9]. All patients that were able to access adjuvant treatments underwent concomitant chemoradiation therapy with 6 weeks from surgery. Time from surgery to first-line therapy was recorded.

All patients were followed-up with brain MRI with gadolinium every 3 months unless new neurological events occurred. Dedicated neuro-oncologists and radiotherapists were involved in treatment and follow-up of our patients. Tumor progression was diagnosed using the RANO criteria.

### 2.6. Statistical Analysis

Categorical variables were described by counts and percentages, and quantitative characteristics were expressed as median (I–III quartiles) or mean and standard deviation (SD), as appropriate. Patients were classified according to the use of operative microscope or exoscope and baseline characteristics (pre-surgery) in these groups were compared using the χ^2^ test or Fisher test for categorical variables and the Mann–Whitney U test for continuous data. Progression-free survival (PFS) was calculated as the time between initial surgery and progressive disease or death, whichever came first. Overall survival (OS) was computed as the time from the first surgery to death, or to the last available follow-up for surviving patients.

Kaplan–Meier method was used to estimate PFS and OS overtime. Log-rank test was applied to compare PFS and OS in the two groups (use ofoperative microscope versus exoscope) and for each relevant clinical characteristic. Due to the recent introduction of the exoscope, in the comparison among the two instruments we artificially censored the follow-up at 12 months (administrative censoring) to ensure homogeneous follow-up.

After checking for the assumption of proportional hazards, a Cox regression model was used to compare the PFS with the use of the two instruments after adjusting for clinical and demographic variables (sex, age, KPS, GTR, MGMT, and the operator’s experience). Two sensitivity analyses were performed to assess robustness of the results: one including the complete follow-up of the patients (without the administrative censoring at 12 months) and the other one including only the patients with initial surgery after 2021. First type error was set at 0.05. All analyses were conducted using R (version 4.0.3).

## 3. Results

### 3.1. Study Population

**Figure 1:**
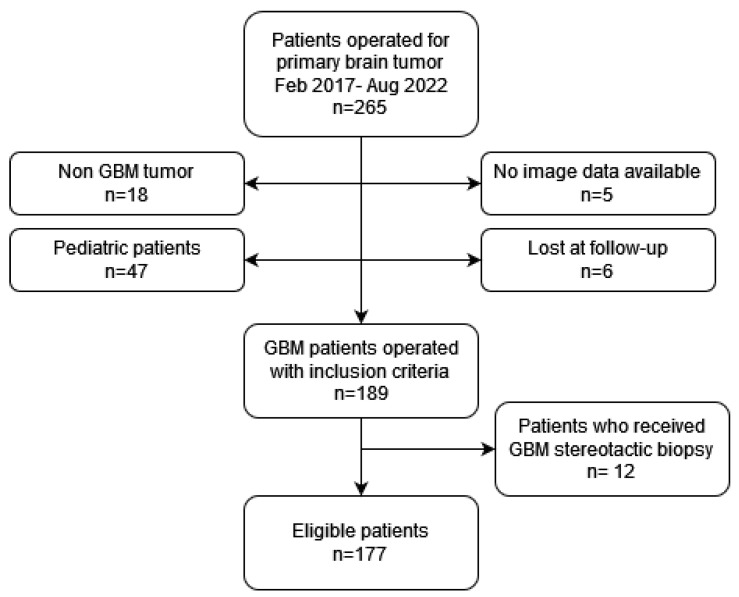
Summary of inclusion and exclusion criteria.

A total of 265 patients were operated for a primary brain tumor during the study period. Of these, 189 (71.3%) patients met the inclusion criteria and were therefore analyzed. A total of 177 (66.8%) patients received surgical resection of the tumor, the remaining 12 patients received open or stereotactic biopsy and were therefore excluded. A flow chart of patient inclusion is shown in Figure 1.

Descriptive characteristics overall and between the two groups are presented in Table 1. The median age was 65 years (I–III quartile = 57–73 years), with no major differences between the two groups (62 years in the exoscope group and 65 years in the microscope group, *p* = 0.139). There were 111 (62.7%) male patients. KPS pre-radiotherapy was equal to or above 70 in 116 patients (67.8%), 26 in the exoscope group and 90 in the microscope group.

Regarding tumor location, 53 lesions (29.9%) were located in the frontal lobe, 53 (29.9%) in the temporal lobe, 24 (13.6%) in the parietal lobe, 24 (13.6%) in the occipital lobe, 11 (6.2%) in the insular lobe, 5 (2.8%) in the corpus callosum, 5 (2.8%) in the thalamus, and 2 (1.1%) lesions were multifocal. Overall, 92 tumors (52%) were located in the right hemisphere, 78 (44.1%) in the left hemisphere, 3 (1.7%) in the midline, and 4 (2.3%) were multifocal.

Taking into account pre-operative symptoms, 22 (11.6%) patients came to our hospital with headache, 25 (13.2%) patients presented with epilepsy, 54 (28.6%) patients came with different degrees of aphasia; 52 (27.5%) patients presented with different degrees of hemisyndrome(mild to severe), 35 (18.5%) patients presented with visual alterations such ashemianopsia or quadrantopsia, and 8 patients presented with no symptoms. A total of 51 (28.8%) patients presented with multiple symptoms.

The median gross tumor volume (GTV) was 41.79 cm^3^. Among patients who received resection surgery, a total of 122 (73.9%) patients received GTR, while 14 (8.5%) patients received STR and 41 (24.8%) patients received PR. No specific complications related to the use of the exoscope were recorded nor was there a need for conversion to the standard microscope.

Histological diagnosis was GBM according to WHO 2021 classification in all cases. IDH was mutated in 9 (5.2%) patients, 4 in the exoscope group and 5 in the microscope group. MGMT was methylated in 76 (44.4%) patients, 10 in the exoscope group and 66 in the microscope group, while in others it was unmethylated.

### 3.2. Overall Outcome

**Figure 2:**
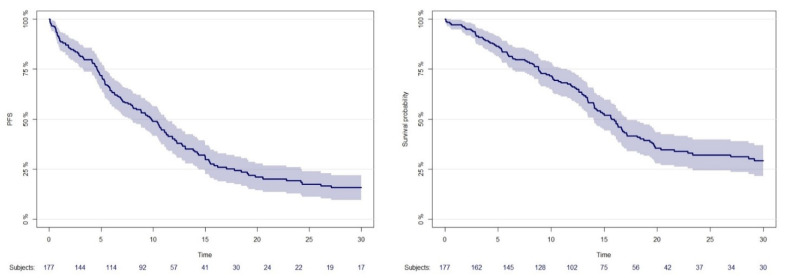
Progression-free survival (PFS) in left part of the figure and overall survival (OS) in overall population in the right part of the figure.

With a median follow-up of 13.43 months, 130 progressions and 17 deaths without progression (for non-tumor related causes) were observed. The overall PFS is reported in Figure 2 (left panel), with a median of 9.83 months (95%CI 8–11.43). Overall OS is reported in Figure 2 (right panel) with a median of 15.70 months (95%CI 14–18). Survival and PFS by relevant characteristics were in line with the literature and are shown in Supplementary Materials (Figure S1).

A total of 165 patients accessed adjuvant therapies; 24 patients had no chemo or radiotherapy. First-line treatment included concomitant chemo-radiation therapy; a total number of 60 patients received a second-line of chemotherapy with regorafenib or fotemustine or bevacizumab, only one patient was treated with carboplatin, and one with thalidomide.

During the neuro-oncological follow-up, a total number of 139 (73.5%) patients experienced tumor recurrence, of which 26 (18.7%) received a second surgery.

### 3.3. Exoscope and Microscope Comparison

Patients operated on with the exoscope had a median GTV of 41.43 cm^3^ while patients operated on with the microscope had a median GTV of 41.88 cm^3^. There were no differences in the preoperative tumor volume between the two groups (*p* = 0.846). IDH was mutated in four patients operated on with the exoscope and in five patients operated on with the operative microscope. The median follow-up for patients operated on with the exoscope was 11.2 months; the median follow-up for patients operated on with the microscope was 29.6 months due to the later introduction of the exoscope.

### 3.4. PFS

**Figure 3:**
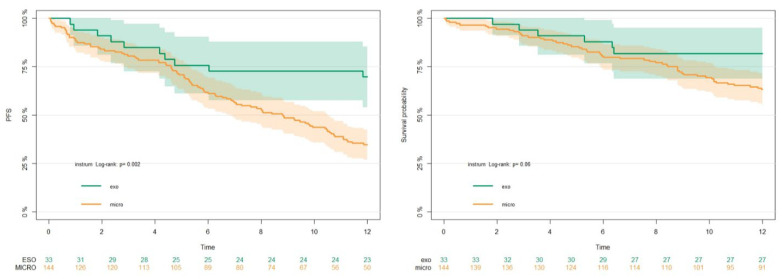
Progression-free survival (PFS), in the left panel, and overall survival (OS), in the right panel, compared between the two instruments.

After truncation to 12 months, PFS was found to be significantly higher in patients operated on with the exoscope (6 months PFS = 75.76%, 95%CI 62–92% and 12 months PFS = 69.7%, 95%CI 55.7–87.3%) when compared to patients operated on with the microscope (6 months PFS = 61%, 95%CI 53–70% and for 12 months PFS = 34.7 95%CI = 27.7–43.4%) (*p* = 0.002) [Figure 3 left]. The median PFS for patients operated on with the microscope was 8.82 months, while for patients operated on with the exoscope it was >12 months.

### 3.5. OS

Overall survival was comparable in the two groups (6 months OS in patients operated on with the exoscope = 87.9%, 95%CI 77–99% and 12 months PFS = 81.8%, 95%CI 69.7–96%, versus 6 months = 79.8%, 95%CI 74–87% in patients operated on with the microscope, 12 months = 63.1, 95%CI 55.8–71.6%, *p* = 0.06) [Figure 3, right].

### 3.6. Cox Model

**Figure 4:**
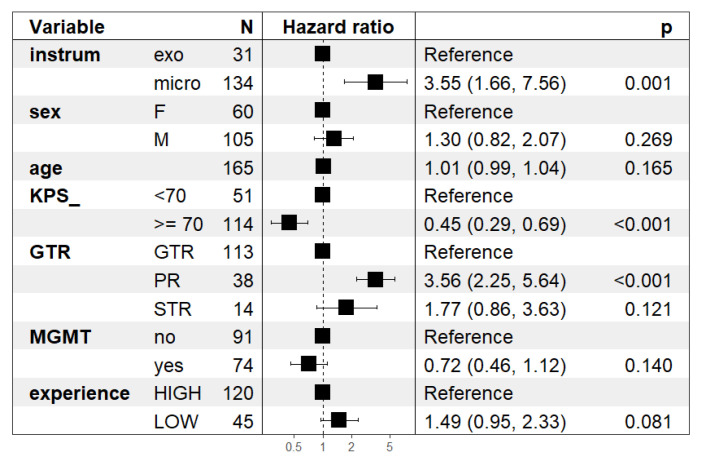
Results of the Cox regression model on progression-free survival with administrative censoring at 12 months. The **left** panel reports the name of the variables included in the Cox model and the number of patients (*n*) in each category. The other two panels report the estimated HR and their95% confidence intervals: the **middle** panel shows a graphical representation as a forest plot; the *p*-values of comparisons are in the **right** panel.

Adjusting for potential confounders, the Cox model showed that the use of microscope compared to the exoscope was associated with lower progression-free survival (hazard ratio, HR = 3.55, 95%CI 1.66–7.56, *p* = 0.001, Figure 4). KPS equal to or higher than 70 was related to better outcome (HR = 0.45, 95%CI 0.29–0.69; *p* < 0.001). GTR showed no statistically significant difference when compared to STR (HR = 1.77, 95%CI = 0.86–3.63; *p* = 0.110), while PR showed worse prognosis (HR = 3.56, 95%CI 2.25–5.64; *p* < 0.001). No statistically significant differences were found for the MGMT methylated promoter (HR = 0.72, 95%CI 0.46–1.12, *p* = 0.140). There were no differences in PFS between patients operated on by surgeons with high or low experience (HR = 1.49, 95%CI 0.95–2.33; *p* = 0.08), for male patients (HR = 1.3, 95%CI 0.82–2.07; *p* = 0.269), or for age (HR = 1.01 for each year of age, 95%CI 0.99–1.04; *p* = 0.165). Of note, the results for the comparison between the two instruments were consistent in the sensitivity analyses with complete follow-up (no administrative censoring at 12 months) and when including only patients with initial surgery after 2021 (Supplementary Materials, Figures S2 and S3).

## 4. Discussion

The use of an exoscope in neurosurgery has been increasing in recent years owing to the uptake of this technology [19,21]; however, only a few authors have compared the operative microscope with the exoscope [21,25,26]. Many experiences have been reported using a 2DHD exoscope that does not allow the perception of depth and stereopsis [27]. The development of an updated technology with introduction of 3D 4K monitors overcame the limits of the first 2D exoscopes [20,28,29]. However, there are still some differences between the standard operative microscope and the exoscope. The improvement inintra operative ergonomics has been reported to be the main positive feature of the exoscope that is able to reduce the surgeons’ intraoperative stress and sitting discomfort [30,31];however, reduced image quality in cases of deep-seated lesions has also been reported [32].

The handling, flexibility, and possibility to easily modify angles of vision during surgery have also been reported as additional positive qualities that overcome the reduced maneuverability of the standard operative microscope [18,33]. In fact, in some cases, vision with the operative microscope cannot reach the closed angles of view as it is impossible to rotate the microscope it self upward [34]. While in case of the exoscope, it can reach a wide variety of angles of view due to the light weight of the camera and its small dimensions [35]. Such features, in contrast with the standard neurosurgical microscope, make the exoscope the type of technology able to change the current microsurgical paradigm. The exoscope offers the opportunity for better interaction in the operative room with collaborators such as nurses and staff, providing a radical change in the paradigm: not only the primary surgeon, but the entire operative room, can be completely involved during surgery [25,36].

In particular, in the case of cranial neurosurgery, the use of the exoscope has been widely described for the surgical treatment of brain tumors, both in adult and pediatric patients [20,30,37,38,39,40], and only few papers have reported a comparison between the operative microscope and the exoscope for brain tumors [26,41,42]. In the study of Takahashi [43], published in 2018, 14 microneurosurgical procedures were retrospectively assessed by 9 neurosurgeons after the procedure by a questionnaire comparing the exoscope (ORBEYE™) to the standard operative microscope. The main benefits of the exoscope in these procedures were reported to be the compact size and freedom of focusing.

However, the most important advantages are related to higher image quality, wider field of view, and a greater sense of depth [27,44]. Gassie et al., in 2018, described the importance of using a 3D viewer exoscope in identifying the sense of depth of surgical field during minimally invasive tubular retractor-assisted resection of deep-seated gliomas [41]. The exoscopic approach allowed visualization of the depth of surgical area without compromising the ergonomics of surgeons [20,41,44,45].

### 4.1. Surgical Resection of Gliomas: Implication of Introduction of a 3D Exoscope

Since the introduction of neuronavigation and brain MRI, glioma surgery requires technologically integrated operating rooms. In recent years, improving technologies has been shown to be necessary to improve neuro-oncological outcome of patients with both low- and high-grade gliomas [14,42,46,47,48]. In the modern neurosurgical era, operative microscope, neuronavigation, and neurophysiological monitoring are standard surgical adjuncts that have been proved to maximize surgical resection, reduce post-operative complications, and neurological deficits [49,50,51,52,53,54,55]. In conjunction, fluorescence-guided surgery with 5ALA and intraoperative imaging, such as intraoperative MRI and intraoperative ultrasound (ioUS), are technologies that have increasingly demonstrated their potential and that will probably become the surgical standard for brain neuro-oncology [46,47,56,57,58,59,60,61,62]. Moreover, the introduction of new technologies has generally been associated with better neurosurgical results in terms of EOR, post-operative complications, and consequently of OS [4,60,63].

Currently, the exoscope represents a new frontier in neurosurgery that is under investigation in order to understand if the introduction of a new microsurgical technology will change the surgical paradigm. In a paper by Calloni et al. [21,36], published in 2023, it was reported that the exoscope seems to be more suitable for training and teaching without differences in terms of the time required for identifying anatomical structures on the operative microscope and the exoscope in anatomical models. A better workflow seemed to be associated with the use of the exoscope [26], although the perception of depth seemed to be the most important limitation of such an instrument [42,64]. In a paper by Strickland et al., it was found that the exoscope seemed to be more useful for brain tumor surgery, although they found the need to switch to the conventional operative microscope in case of deep surgical fields [48].

Other reported disadvantages encompassed some difficulties for the assisting neurosurgeon due to the reversed surgical field on the monitors [43]. This limitation might be particularly significant in cases of teaching (the senior tutor may experience some difficulties in aiding or guiding a trainee) or in the case of large bleeds that may require some help by the assisting neurosurgeon in order to keep the surgical field clear.

For these reported reasons, Murai et al. [65] describe the impossibility of substituting the microscope in all neurosurgical procedures, even if the exoscope surpasses the microscope in terms of ergonomic features.

However, only few studies assessed the non-inferiority of a 3D exoscope when compared to a standard binocular microscope, and none have compared GBM patients in terms of clinical, surgical, and survival outcome. Most of the studies were focused on the use of 5ALA with the exoscope [20,26,38,39,44,65,66,67], including operative videos [38,42,66] and descriptive studies [29,48,64,68,69]. No studies involved a direct comparison with the standard operative microscope except for one study by Roethe et al., which concluded that the 3D exoscope was useful for superficial brain tumors; however, the study was a qualitative assessment of the exoscope in terms of practical tasks, with no focus on patient outcome [70].

Few studies have analyzed the difference in extent of resection of glioma between the two instruments. In particular, a case report of Strickland et al. [48] described the shift from microscope to exoscope for the resection of an eloquent area GBM with the evaluation of the EOR as >98%. Thestudy of Piquer et al. [69] evaluated the EOR in 38 patients with high-grade glioma, of which 23 received GTR and 7 received STR.

Despite the increasing literature and interest on this instrument, only few studies assess the safety of the exoscope compared to the microscope [20,39,48,68] and no study, apart from several case reports [20,26,68], assesses the comparison in terms of surgical outcome, clinical outcome, and survival outcome for GBM patients.

### 4.2. Our Experience

The experience of IRCCS Fondazione San Gerardo deiTintori began in the 2021 with the introduction of an exoscope for major surgeries; before this, only the standard operative microscope was available. In our experience, the exoscope rapidly became the instrument of choice for tumor surgery. The major advantage was the compact size in contrast with modern bulky microscopes. The learning curve for both leading surgeons and assistant neurosurgeons was steep, even if challenging at the beginning. The first related difficulty was the main screen positioning: even if the main screen is always positioned in front of the first surgeon, the second screen must accommodate the vision angle of both the second operator and the nurse in a highly technological operative room, already occupied by equipment for neuronavigation, ultrasonic aspirator, ioUS, and intraoperative neurophysiological monitoring. However, this difficulty was overcome by applying a multidisciplinary balance before the beginning of each surgery in order to position all the screens in the best way for all the team. During surgery, the exoscope proved its feasibility for both superficial and deep approaches especially in cranial surgery. However, in deep surgical fields, we often found imprecision in the autofocus that necessitated manual adjustment.

In our experience of GBM, surgery with the exoscope was characterized by the perception of a steep learning curve for all surgeons. Statistical analysis revealed a study population comparable with the current literature in terms of survival, surgical, and neuro-oncological outcomes at a GTR rate of 73.9%. In specific analyses, no differences were found between the two instruments in terms of patients’ pre-operative characteristics, EOR, or postoperative deficits. Patients operated on with the exoscope had a better PFS (*p* = 0.002), but not OS (*p* = 0.06), than patients operated on with the standard microscope. This may suggest that the use of a 3D4K exoscope can contribute to maximize the EOR, although no comparisons were made due to the high rate of GTR among patients operated on with the exoscope. No complications related to the use of the exoscope were recorded. Furthermore, in models adjusted with covariates such as sex, KPS, age, percentage of resection, and molecular analysis, patients operated on with the exoscope had better progression-free survival than patients operated on with the microscope (hazard ratio HR = 3.55, 95%CI 1.66–7.56, *p* = 0.001).

Taken together, our results suggest that the exoscope may offer a better PFS due to the high rate of GTR, which is likely related to the wider view that the exoscope can offer. In our opinion, the exoscope can give a global view of the surgical field, while the microscope can offer a focused view. A focused view may be associated with a less aggressive perilesional resection that may confer a higher risk for a short PFS.

In conclusion, the exoscope is safe and feasible for patients with GBM; our case series demonstrated a comparable surgical and clinical outcome for the two instruments, and a better PFS outcome for patients operated on with the exoscope. However, these results must be considered with caution, as this is the first study, to our knowledge, comparing patients operated on for GBM with an exoscope or microscope.

### 4.3. Limitations of the Study

This study has some limitations. First of all, the study is observational and thus we cannot draw any causal relationship between exposure and outcome. Moreover, the population encompassed a larger sample of patients operated on with the microscope compared with a smaller number of patients operated on with the exoscope in the most recent period (2021–2022). To overcome this last issue, we performed a sensitivity analysis on the most recently operated patients and found consistent results. However, our results may change in further studies that will include a wider group of patients operated on with the exoscope.

Moreover, due to the recent introduction of the exoscope in our division, the follow-up of patients operated on for GBM is relatively short. Interestingly, the PFS of patients operated on with the exoscope was higher than the PFS of those operated on with the microscope; additionally, when the follow-up was cut to be comparable among the two groups, suggesting that the introduction of a new technology helped to increase the EOR. In contrast, this result may be due to the small sample size of patients operated on with the exoscope included in the study (*n* = 33) considering that, in both groups, nearly 80% of the cases received GTR.

A future study with longer follow-up is necessary in order to better analyze differences in OS or PFS even for long-surviving patients.

## 5. Conclusions

To our knowledge, this is the first study in the literature that compares the microscope and the exoscope in terms of outcome in GBM patients. The efficacy of the exoscope was proven in terms of surgical resection, which was not different to the microscope group. Furthermore, postoperative clinical outcomes were comparable and patients operated on with the exoscope did not have more neurological deficits when compared to the microscope group. Similar survival outcomes were assessed between two groups. However, as new technologies over time improve survival for oncology patients, our results suggest that the exoscope could ensure a longer progression-free and, probably overall survival, for GBM patients. If this result can be confirmed in further studies, this could be the beginning of a new paradigm change in neuro-oncological surgery.

## Figures and Tables

**Table 1:** Descriptive features of study population.

	Overall	EXOSCOPE	MICROSCOPE	*p*
*n* (%)	177	33 (18.6%)	144 (81.4%)	
**AGE** (**median I–III**)	65 (57,73)	62 (51, 73)	65 (58, 73)	0.139
**SEX = M, *n*** (**%**)	111 (62.7)	23 (69.7)	88 (61.1)	0.471
**Experience = LOW, *n*** (**%**)	47 (26.6)	11 (33.3)	36 (25.0)	0.448
**LOBE, *n*** (**%**)				0.749
Frontal	53 (29.9)	11 (33.3)	42 (29.2)	
Temporal	53 (29.9)	9 (27.3)	44 (30.6)	
Insular	11 (6.2)	4 (12.1)	7 (4.9)	
Occipital	24 (13.6)	4 (12.1)	20 (13.9)	
Parietal	24 (13.6)	4 (12.1)	20 (13.9)	
Corpus callosum	5 (2.8)	0 (0.0)	5 (3.5)	
Thalamus/pineal	5 (2.8)	1 (3.0)	4 (2.8)	
Multifocal	2 (1.1)	0 (0.0)	2 (1.4)	
**SIDE, *n*** (**%**)				0.846
Right	92 (52.0)	17 (51.5)	75 (52.1)	
Midline	3 (1.7)	0 (0.0)	3 (2.1)	
Multifocal	4 (2.3)	1 (3.0)	3 (2.1)	
Left	78 (44.1)	15 (45.5)	63 (43.8)	
**IDH = Mutation, *n*** (**%**)	9 (5.2)	4 (12.1)	5 (3.6)	0.120
**MGMT = Methylation, *n*** (**%**)	76 (44.4)	10 (30.3)	66 (47.8)	0.104
**GTR, *n*** (**%**)				0.148
GTR	122 (68.9)	23 (69.7)	99 (68.8)	
PR	41 (23.2)	5 (15.2)	36 (25.0)	
STR	14 (7.9)	5 (15.2)	9 (6.2)	
**KPS ≥ 70, *n*** (**%**)	116 (67.8)	26 (83.9)	90 (64.3)	0.057

Abbreviation: GTR = gross total resection; STR = subtotal resection; PR = partial resection.

## Data Availability

The data presented in this study are available on request from the corresponding author. The data are not publicly available due to ethical restrictions.

## Supplementary Materials

The following supporting information can be downloaded at: https://www.mdpi.com/article/10.3390/brainsci13071035/s1, Figure S1: PFS and OS for clinical characteristics; Figure S2: Results of Cox model with sensitivity without administrative censoring; Figure S3: Results of Cox model considering including only patients with surgery after 2021.
