# Supplementary material for: Exoscopic Microsurgery: A Change of Paradigm in Brain Tumor Surgery? Comparison with Standard Operative Microscope
Source: Brain Sci. 2023 Jul 6;13(7):1035. doi: 10.3390/brainsci13071035 (PMC10377370; doi:10.3390/brainsci13071035)

SUPPLEMETARY MATERIALS

Exoscopic Microsurgery: a Change of Paradigma in Brain Tumors Surgery? Comparisons with standard operative microscope

Andrea Di Cristofori,Francesca Graziano, Chiara Benedetta Rui, Paola Rebora, Diego Di Caro, Gaia Chiarello, Giovanni Stefanoni, Chiara Julita, Santa Florio, Davide Ferlito, Gianpaolo Basso, Giuseppe Citerio, Paolo Remida, Giorgio Carrabba and Carlo Giussani

Figure S1 - PFS and OS for clinical characteristics

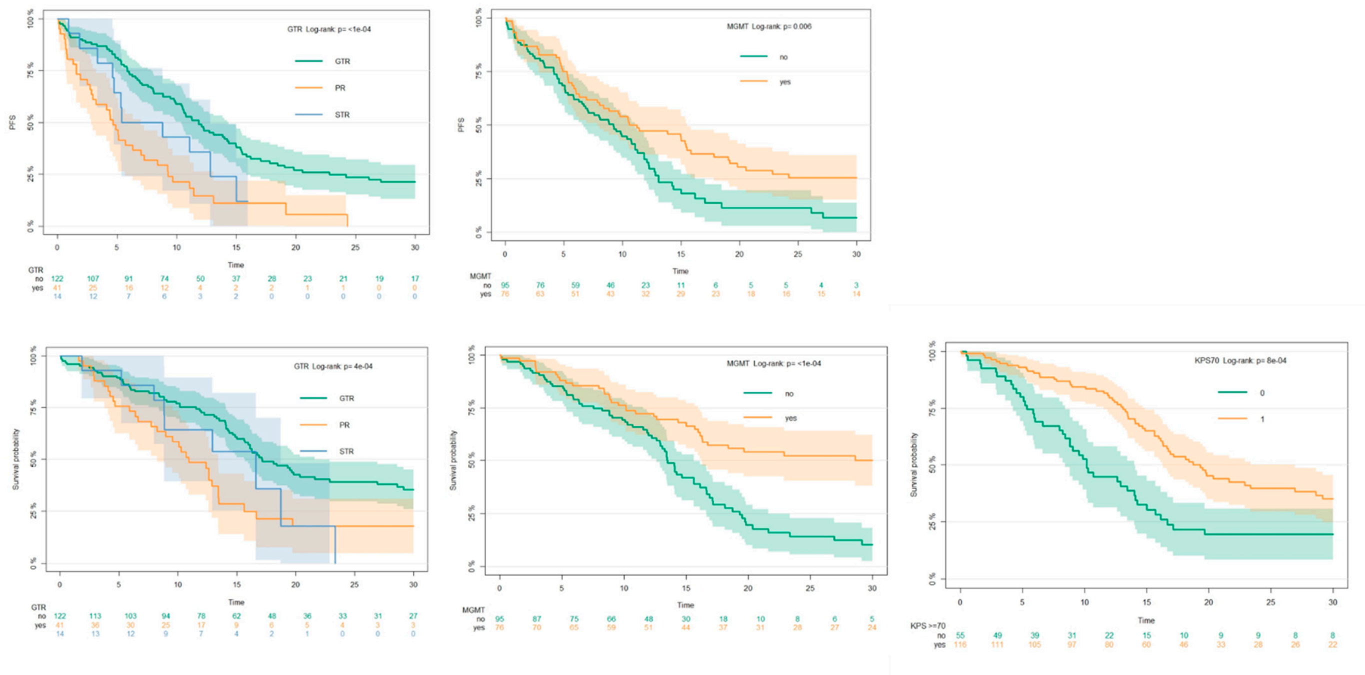

Figure S2 - Results of Cox model with sensitivity without administrative censoring

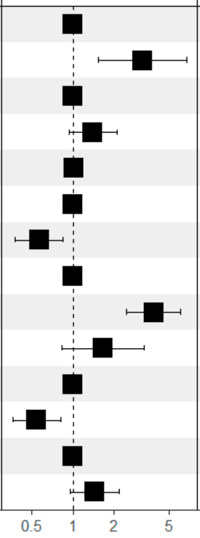

Variable		N	Hazard ratio		p
instrum	exo	31		Reference	
	micro	134		3.20 (1.52, 6.75)	0.002
sex	F	60		Reference	
	M	105		1.40 (0.94, 2.07)	0.098
age		165		1.01 (0.99, 1.03)	0.163
KPS_	<70	51		Reference	
	>= 70	114		0.57 (0.38, 0.85)	0.006
GTR	GTR	113		Reference	
	PR	38		3.88 (2.46, 6.12)	<0.001
	STR	14		1.66 (0.84, 3.29)	0.148
MGMT	no	91		Reference	
	yes	74		0.54 (0.36, 0.81)	0.003
experience	HIGH	120		Reference	
	LOW	45		1.43 (0.95, 2.15)	0.087

Figure S3 - Results of Cox model considering including only patients with surgery after 2021

Variable		N	Hazard ratio		p
**instrum**	exo	31		Reference	
	micro	32		4.79 (2.01, 11.46)	<0.001
**sex**	F	23		Reference	
	M	40		1.56 (0.64, 3.77)	0.32
**age**		63		1.01 (0.97, 1.06)	0.51
**KPS_**	<70	17		Reference	
	>= 70	46		0.35 (0.16, 0.78)	0.01
**GTR**	GTR	43		Reference	
	PR	13		2.55 (1.03, 6.30)	0.04
	STR	7		1.28 (0.34, 4.80)	0.71
**MGMT**	no	38		Reference	
	yes	25		0.59 (0.26, 1.34)	0.20
**experience**	HIGH	43		Reference	
	LOW	20		0.75 (0.33, 1.71)	0.49

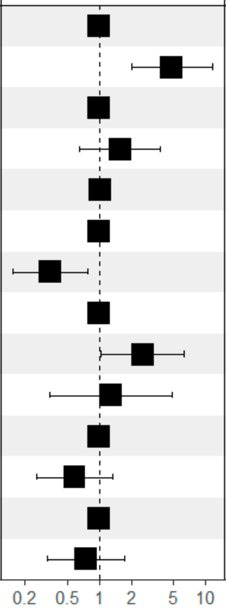

Supplement: Supplementary file 1 [file brainsci-13-01035-s001.zip › brainsci-2479169-supplementary.pdf]
